# Cryopreservation of primary B cells minimally influences their signaling responses

**DOI:** 10.1038/s41598-018-36121-9

**Published:** 2018-12-05

**Authors:** Johanne U. Hermansen, Geir E. Tjønnfjord, Ludvig A. Munthe, Kjetil Taskén, Sigrid S. Skånland

**Affiliations:** 1 Centre for Molecular Medicine Norway, Nordic EMBL Partnership, University of Oslo and Oslo University Hospital, Oslo, Norway; 2 Department of Haematology, Oslo University Hospital, Oslo, Norway; 3 Institute of Clinical Medicine, University of Oslo, Oslo, Norway; 4 K. G. Jebsen Centre for B Cell Malignancies, University of Oslo, Oslo, Norway; 5 Department of Immunology, Oslo University Hospital, Oslo, Norway; 6 K. G. Jebsen Centre for Cancer Immunotherapy, University of Oslo, Oslo, Norway; 7 Department of Cancer Immunology, Institute for Cancer Research, Oslo University Hospital, Oslo, Norway

## Abstract

Phospho flow is a powerful approach to detect cell signaling aberrations, identify biomarkers and assess pharmacodynamics, and can be performed using cryopreserved samples. The effects of cryopreservation on signaling responses and the reproducibility of phospho flow measurements are however unknown in many cell systems. Here, B lymphocytes were isolated from healthy donors and patients with the B cell malignancy chronic lymphocytic leukemia and analyzed by phospho flow using phospho-specific antibodies targeting 20 different protein epitopes. Cells were analyzed both at basal conditions and after activation of cluster of differentiation 40 (CD40) or the B cell receptor. Pharmacodynamics of the novel pathway inhibitor ibrutinib was also assessed. At all conditions, fresh cells were compared to cryopreserved cells. Minimal variation between fresh and frozen samples was detected. Reproducibility was tested by running samples from the same donors in different experiments. The results demonstrate reproducibility across different phospho flow runs and support the use of cryopreserved samples in future phospho flow studies of B lymphocytes.

## Introduction

Cancer represents an extremely heterogeneous disease underscored by high genetic variability, risk of clonal progression of resistant tumors after treatment with targeted therapies and diverse clinical outcomes. Much effort is invested in identification of biomarkers showing prognostic value, indicating treatment options or predicting disease outcome. Novel targeted therapies are often directed at signaling molecules, including the PI3Kδ inhibitor idelalisib and the Bruton tyrosine kinase (BTK) inhibitor ibrutinib, both used to treat B cell malignancies, including chronic lymphocytic leukemia (CLL)^1^. Since activation of mitogenic signaling pathways play a central role in cancer development and progression it is reasonable to assume that the activity level of central signaling molecules may serve as cancer biomarkers. For medium- to high-throughput screening of single cell phospho-protein levels, phospho flow cytometry is an attractive approach^2^. By applying this technique, signaling patterns have been characterized in diverse B cell malignancies^3–6^. We recently identified STAT3 (pY705) as aberrantly upregulated in a group of CLL patients and showed that STAT3 inhibitors potently kill the cancer cells^4^. Phospho flow analysis can thus be used to identify relevant drug targets.

Characterization of cell signaling is commonly performed on cryopreserved cells. Cryopreservation allows long-term storage of lymphocytes before immunological assays are performed. In this way, samples collected at multiple time points or from multiple patients can be analyzed simultaneously, reducing variation arising from different runs. Frozen samples can also easily be transported to a single laboratory for analysis, reducing variation introduced by different operators or laboratory conditions^7^. This is particularly useful in multi-center trials when fresh samples cannot be transported within an acceptable time frame to ensure intact samples. It is, however, important that freezing, storage and thawing do not have major negative impact on assay read-outs.

The effects of cryopreservation have been most extensively studied in T cells, but opinion and empirical evidence are divided. To varying degree cryopreservation has been shown to alter stability of surface marker expression^8–10^, cytokine production^9^ and cell proliferation^11^. Such discrepancies can, however, be minimized when an optimized protocol is followed^10,12–14^. Freezing of cells within 12 h of blood collection^12^, minimized temperature fluctuations during storage^15^ and resting of the thawed cells before staining for phenotypic analysis^16^ are factors which have been shown to improve preservation of cell function. In general, T cells have been shown to be more sensitive to cryopreservation than other immune cells^16^. Studies on the effect of cryopreservation on B-cell function by ELISpot have shown little variation between fresh and frozen cells and thus support the use of cryopreserved samples^7,17^.

The aim of this study was to compare signaling responses in B lymphocytes detected by phospho flow using both fresh and frozen samples and to test the reproducibility of the phospho flow assay.

## Methods

### Patient material and ethical considerations

Buffy coats from anonymized healthy blood donors and blood samples from CLL patients were received from the Blood Centre (Oslo University Hospital) and the Department of Haematology, Oslo University Hospital, respectively, following written informed consent from all donors. The study was approved by the Regional Committee for Medical and Health Research Ethics of South-East Norway, and the research on human blood was carried out in accordance with the Declaration of Helsinki^18^.

### Reagents and antibodies

sCD40L (cat. no. 11343345) was from ImmunoTools, Germany and F(ab’)2 anti-human IgM (cat. no. 2022-01) was from Southern Biotechnology, CA, USA. Ibrutinib was purchased from Selleckchem (TX, USA). A list of the antibodies used in this study can be found in Supplementary Table 1.

### Isolation of B lymphocytes

Normal B cells were isolated from buffy coats by negative selection using RosetteSep Human B Cell Enrichment Cocktail (20 μl/mL blood; Stemcell Technologies, Cambridge, United Kingdom) followed by gradient centrifugation with Lymphoprep (Alere Technologies AS, Oslo, Norway). CLL cells were isolated from whole blood by Lymphoprep according to the manufacturer’s protocol. Cell samples were either analyzed immediately after isolation or cryopreserved in liquid nitrogen. Cryopreserved cells were rested in RPMI 1640 GlutaMAX medium supplemented with 10% fetal calf serum at 37 °C and 5% CO_2_ for one hour after thawing.

### Phospho flow with fluorescent cell barcoding

Single cell signaling analysis was carried out using phospho flow with fluorescent cell barcoding as described^19^. Briefly, B cells were treated as described in the figure legends before fixation (BD Phosflow Fix Buffer I, BD Biosciences, Franklin Lakes, NJ, USA). The cells were then washed three times with PBS and stained with different concentrations of the barcoding reagents Alexa Fluor 488 5-TFP, Pacific Blue Succinimidyl Ester and Pacific Orange Succinimidyl Ester (all from ThermoFisher Scientific, Waltham, MA, USA) for 20 min at room temperature. The cells were then washed twice with flow wash (PBS, 10% FCS and 0.09% sodium azide), combined in one tube and permeabilized with BD Phosflow Perm Buffer III (BD Biosciences) which was pre-cooled at −20 °C, and kept at −80 °C until further processing. Before antibody staining, the cells were washed three times with flow wash. The antibodies used in this study are listed in Supplementary Table 1, including the surface marker anti-CD19. After 30 min incubation with the antibodies, the cells were washed once, resuspended in flow wash and analyzed with a BD FACSCanto II (4-2-2) cytometer equipped with 405 nm, 488 nm and 633 nm lasers. The data were analyzed in Cytobank (https://cellmass.cytobank.org/cytobank/) as described^4^.

### Statistical analysis

Statistical analyses were performed in Prism 7 (GraphPad Software, CA, USA) using the statistical tests indicated in the figure legends.

## Results and Discussion

### Characterization of basal signaling patterns in freshly isolated and cryopreserved primary B cells

**Figure 1:**
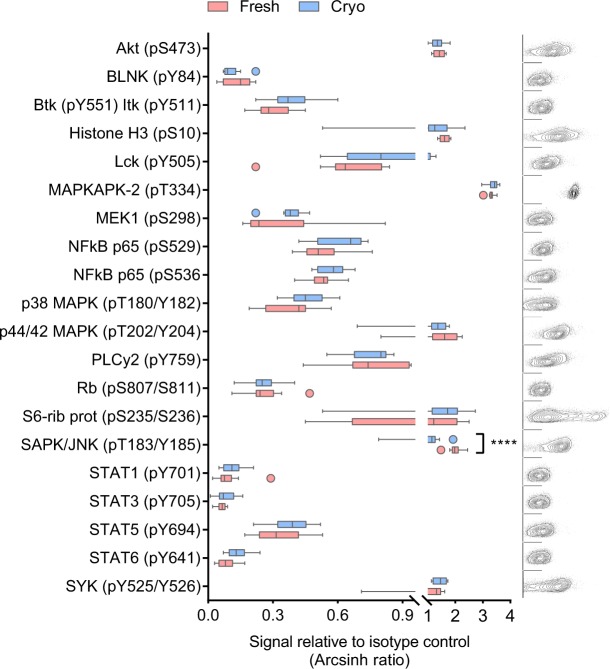
Characterization of basal signaling patterns in freshly isolated and cryopreserved primary B cells. B cells were isolated from healthy blood donors and analyzed immediately (n = 10; fresh) or after cryopreservation (n = 10; cryo). The cells were fixed, permeabilized and stained with anti-CD19 surface marker and phospho-specific antibodies as described in Methods. The fluorescence signals were detected by flow cytometry and analyzed in Cytobank. The basal fluorescence signals were measured relative to IgGκ isotype control and shown as arcsinh ratio (box-and-whisker; outliers are indicated separately). Statistical significance was calculated by a paired t-test with correction for multiple comparisons using the Holm-Sidak’s method, ****p < 0.00001. The plots to the right show raw flow cytometry data from one representative donor (fresh). Each plot corresponds to the protein shown in the aligned box-and-whisker plot, with phospho-protein (x-axis) plotted against FSC-A (y-axis).

In order to investigate whether the basal phosphorylation level in primary B cells is affected by cryopreservation, a medium throughput single cell signaling analysis was carried out on freshly isolated and cryopreserved primary human B cells from 10 healthy donors by phospho flow (Fig. 1). The phosphorylation level of 20 signaling molecules relevant to B-cell signaling was analyzed. As many as 19 out of 20 phospho-proteins were not differently regulated in freshly isolated versus cryopreserved B cells (Fig. 1). This analysis demonstrates that basal signaling patterns in B cells are nearly unaffected by cryopreservation and suggests that cryopreserved cells can serve as a model for fresh cells in studies of basal signaling.

### Phospho flow measurements are highly reproducible

**Figure 2:**
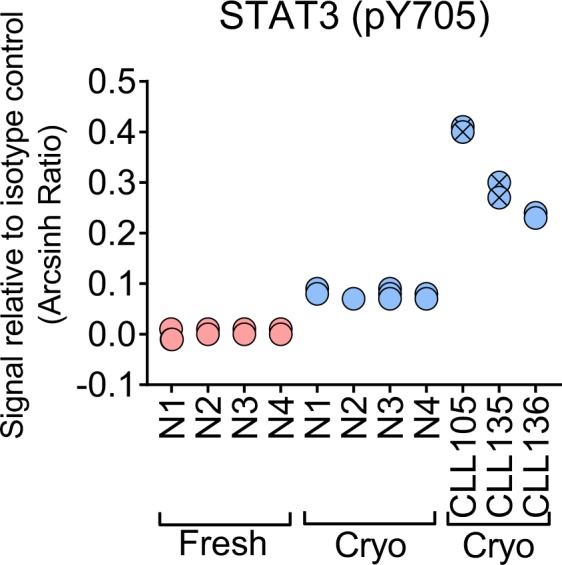
Phospho flow measurements are highly reproducible. Basal STAT3 (pY705) signals were analyzed in normal B cells from four healthy blood donors (N1-N4) before (fresh) and after (cryo) cryopreservation, and in cryopreserved B cells from three CLL patients (CLL105, CLL135, CLL136). Symbols with a cross represent CLL patients with unmutated IgVH status. The analysis was done on three samples from each donor on three different days, following the procedure described in Methods.

Characterization of signaling patterns by phospho flow has shown promising utility in cancer biomarker discovery^20,21^. When patient samples are analyzed in search of predictive biomarkers, small differences in signaling amplitude may distinguish patients classified as positive or negative for a given biomarker. It is therefore very important that the phospho flow assay produces reliable and reproducible data. In order to test the reproducibility of the method, B lymphocytes from four healthy donors were aliquoted and fixed, followed by antibody staining and analysis on three different days. The normal B cells were analyzed for STAT3 (pY705) phosphorylation level both before and after cryopreservation (Fig. 2). As shown, the triplicates presented highly similar, almost overlapping, phospho-signals, demonstrating the robustness of the assay (Fig. 2). A recent report from our group showed that the basal level of STAT3 (pY705) is upregulated in CLL patients^4^. Three cryopreserved CLL patient samples were therefore included as a control and analyzed in triplicate on three different days. The STAT3 (pY705) levels in the triplicate patient samples were strongly reproducible and, in agreement with our previous report, elevated compared to the level in normal B cells (Fig. 2). These results indicate that phospho flow is a reliable method for biomarker identification.

In addition to the low intra-donor variability in phospho-protein levels detected by phospho flow, we here show that the variability between healthy donors, as well as between different studies performed by different operators, is low. Ten cryopreserved healthy samples from an earlier study^4^ were compared with the ten cryopreserved healthy samples from this study. As shown in Supplementary Fig. 1, the basal phosphorylation levels of all 20 proteins analyzed were similar between the two groups. This suggests that a relatively low number of healthy donors is needed to control patient studies and that historical data can be used.

### sCD40L induced signaling is not significantly affected by cryopreservation

**Figure 3:**
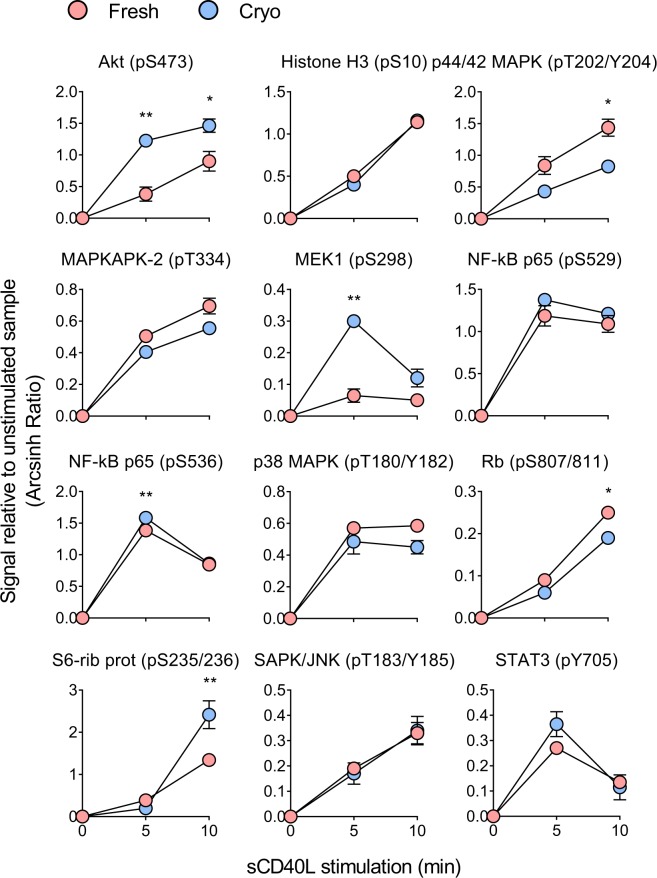
sCD40L induced signaling is not significantly affected by cryopreservation. B cells from the same two healthy donors were analyzed before (fresh) and after (cryo) cryopreservation. The cells were stimulated with sCD40L (400 ng/mL) for 5 and 10 min before fixation and phospho flow analysis as described in Methods. Results are shown as mean ± standard deviation. Statistical significance was calculated between the two groups (fresh, cryo) by an ordinary two-way ANOVA with Sidak’s multiple comparisons test. **p < 0.01, *p < 0.05.

When cells are located in their natural microenvironment they will receive signals from the microenvironment which will induce signal transduction and phosphorylation events. We have shown that activation of the CD40 co-receptor induces distinct signaling patterns in normal B cells and CLL cells^6^. To test if protein phosphorylation induced by microenvironmental stimuli is affected by cryopreservation, phospho flow was performed on fresh and cryopreserved normal B cells after exposure to sCD40L for 5–10 min. Twenty signaling molecules (see Fig. 1) were analyzed, of which 12 that displayed significantly induced phosphorylation levels upon sCD40L stimulation are presented in Fig. 3. Of these, six proteins did not show any significant variation in regulation between fresh and cryopreserved cells. Five proteins showed statistically different phosphorylation level at only one of the time-points tested, whereas Akt (pS473) as the only protein was significantly different in fresh and cryopreserved cells at both time-points (Fig. 3). Interestingly, the proteins that displayed modified signaling response as a result of cryopreservation primarily belonged to the MAPK and PI3K pathways. In summary, little variation is observed between fresh and cryopreserved cells after sCD40L stimulation.

### Signaling downstream of the B-cell receptor is mostly unaffected by cryopreservation

**Figure 4:**
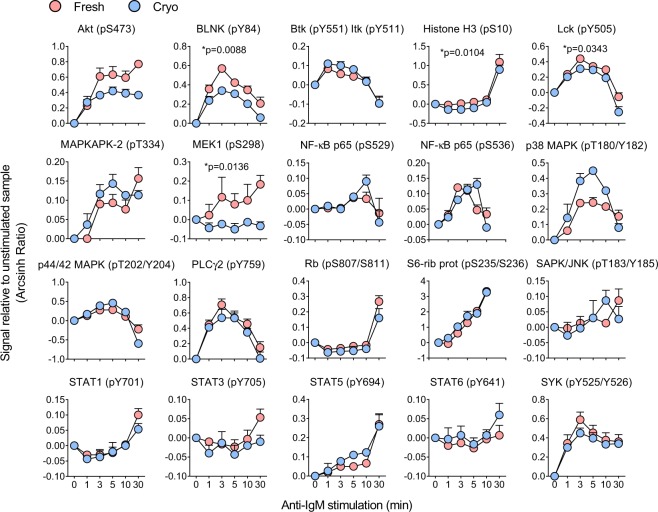
Signaling downstream of the BCR is mostly unaffected by cryopreservation. B cells were isolated from healthy blood donors and analyzed immediately (n = 3; fresh) or after cryopreservation (n = 3; cryo). The cells were stimulated with anti-IgM (10 μg/mL) for the indicated time-course before fixation and phospho flow analysis as described in Methods. Results are shown as mean ± standard error of the mean (SEM). Statistical significance was calculated between the two groups (fresh, cryo) by a paired t-test with excluded time parameter. Significant *p*-values are indicated.

The B-cell receptor (BCR) plays a central role in B cell signaling and development of B cell malignancies. Over the last few years, management of hematological diseases has focused on novel therapies that target the BCR signaling pathway, including the Btk inhibitor ibrutinib^1^. BCR induced signaling is therefore very relevant in biomarker discovery studies. To test if BCR signaling is affected by cryopreservation, normal B cells were stimulated with anti-IgM for up to 30 min. The effects on 20 signaling molecules were monitored (Fig. 4). For the majority of the analyzed proteins the signaling responses in fresh and cryopreserved cells were practically identical (Fig. 4). Only four proteins [BLNK (pY84), Histone H3 (pS10), Lck (pY505) and MEK1 (pS298)] showed statistically significant responses in fresh versus cryopreserved cells. In conclusion, BCR induced signaling is mostly unaffected by cryopreservation.

A similar study of anti-IgM induced signaling responses was previously performed on B cells from 10 healthy donors^4^. Since the data analysis was performed in the same way, and the data are presented as arcsinh ratio in both studies, the effects can easily be compared. As shown in Supplementary Fig. 2, for most of the 20 phospho-proteins analyzed, the signals of the donors from the two studies lie within the same range. This confirms low variability in phospho flow experiments over time and between operators, as observed in other assays on primary B cells^7^.

### Signaling responses induced by ibrutinib treatment are unaffected by cryopreservation

**Figure 5:**
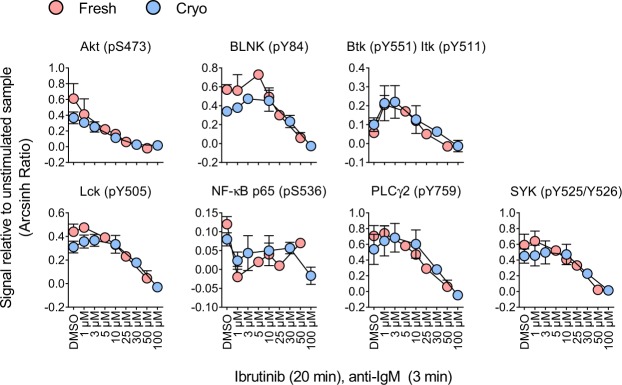
Signaling responses induced by ibrutinib treatment is unaffected by cryopreservation. B cells were isolated from healthy blood donors and analyzed immediately (n = 3; fresh) or after cryopreservation (n = 3; cryo). The cells were treated with the indicated concentrations of ibrutinib for 20 min before stimulation with anti-IgM (10 μg/mL) for three min. The cells were then fixed and analyzed by phospho flow as described in Methods. Results are shown as mean ± standard deviation. Statistics were performed as in Fig. 4. No significant *p*-values were achieved.

Assessment of pharmacodynamics of drugs that interfere with signaling is one application of phospho flow and a central topic in precision medicine. As mentioned above, novel cancer therapies include BCR inhibitors such as ibrutinib. Here, the effect of cryopreservation on pharmacodynamics of ibrutinib was investigated as an example. Normal B cells were exposed to a range of ibrutinib concentrations for 20 min before stimulation with anti-IgM for three min. Ibrutinib treatment reduced the phosphorylation level of several proteins downstream of the BCR (Fig. 5). However, some of the effects were only observed at very high concentrations and are probably not clinically relevant. Importantly, no statistically significant differences in signaling responses were detected between fresh and cryopreserved cells (Fig. 5).

## Conclusion

Minimal variation was observed between fresh and cryopreserved primary B cells in phospho flow assays, which is in agreement with earlier reports in which other methods were applied^7,17^. Importantly, the presented protocol was optimized to improve preservation of cell function after cryopreservation, i.e. the cells were frozen within 12 h of blood collection^12^, temperature fluctuations were minimal during storage^15^ and the thawed cells were rested before signaling analysis^16^. In conclusion, the present study supports the use of cryopreserved cells in biomarker discovery and assessment of pharmacodynamics in B cell malignancies.

## Electronic supplementary material

Dataset 1
