# Supplementary material for: Cryopreservation of primary B cells minimally influences their signaling responses
Source: Sci Rep. 2018 Dec 5;8:17651. doi: 10.1038/s41598-018-36121-9 (PMC6281576; doi:10.1038/s41598-018-36121-9)
Supplement: Supplementary file 1 — Dataset 1 [file 41598_2018_36121_MOESM1_ESM.pdf]

Cryopreservation of primary B cells minimally influences their signaling responses

Johanne U. Hermansen[1], Geir E. Tjønnfjord[2,3,4], Ludvig A. Munthe[4,5], Kjetil Taskén[1,4,6,7], Sigrid S. Skånland[1,4,6,7,*]

SUPPLEMENT

FIGURE LEGENDS

Supplementary figure 1. Basal signaling is comparable between different studies on healthy donors. Comparison of basal phospho-protein levels reported in[4] (Myhrvold *et al.,* n=10) and the present study (Cryo, n=10). See legend to Figure 1 for experimental details. Statistical significance was calculated by a paired t-test with correction for multiple comparisons using the Holm-Sidak's method. No significant *p*-values were identified.

Supplementary figure 2. BCR signaling is comparable between healthy donors and between different studies. Comparison of signaling responses reported in[4] (green symbol; mean of n=10 healthy donors, ± SEM) and in the present study (cryo and fresh, both n=3). See legend to Figure 4 for experimental details.

Supplementary figure 1

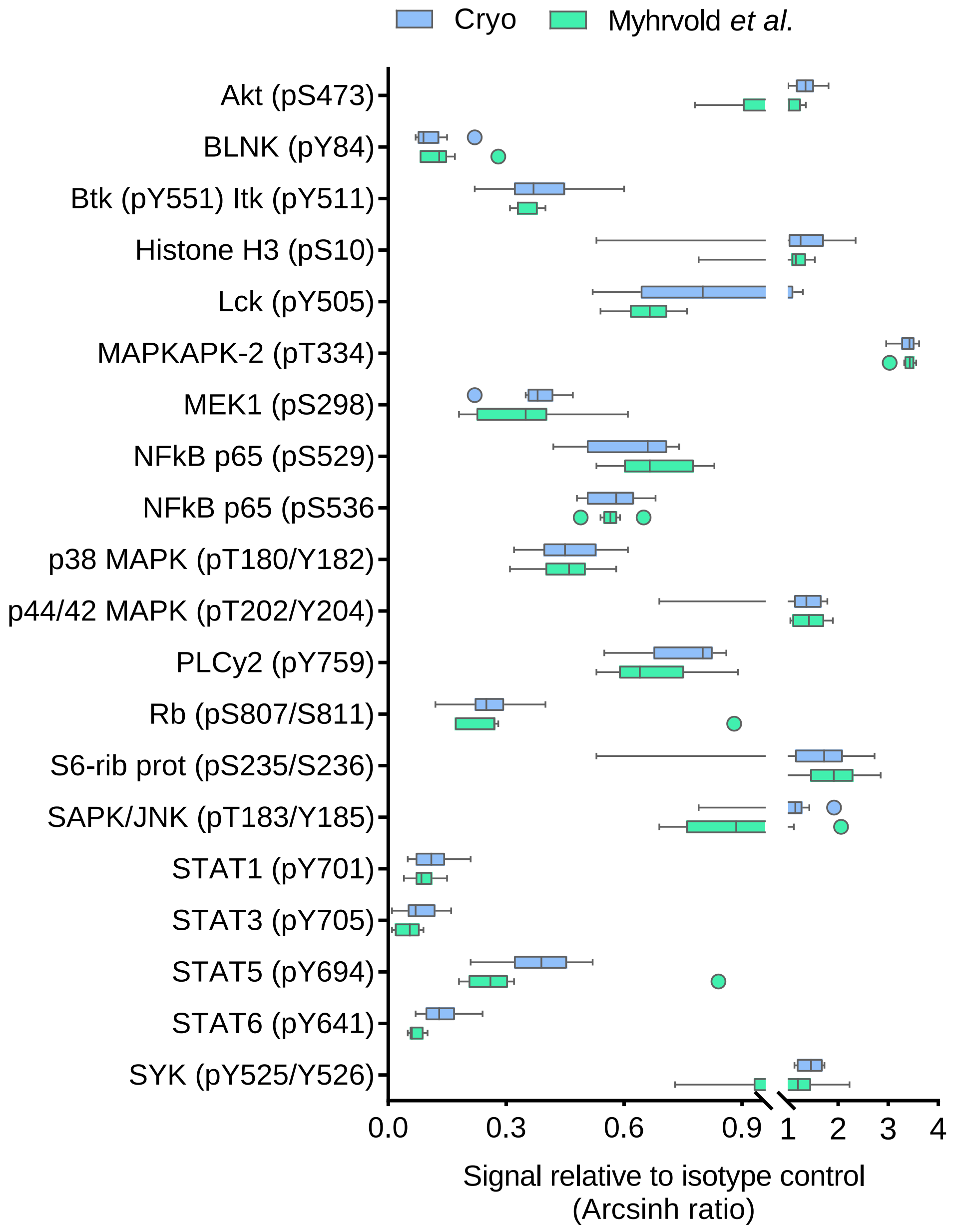

Supplementary figure 2

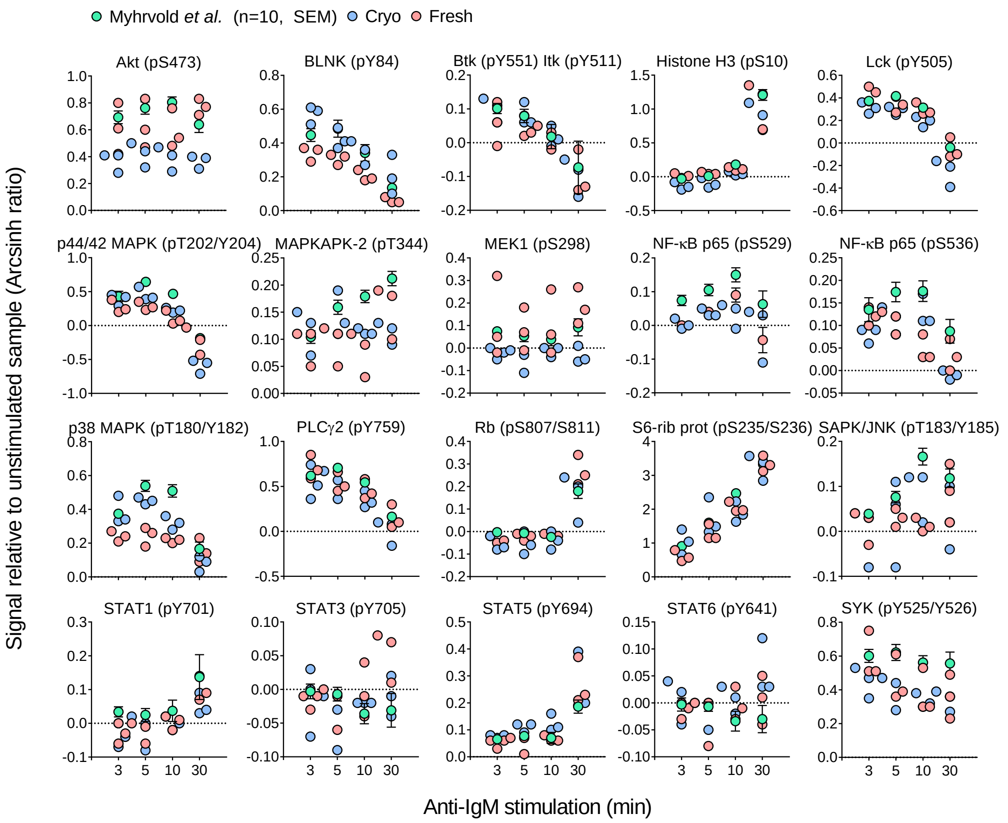

SUPPLEMENTARY TABLE 1: Antibodies used in the study

Antigen	Vendor*	Clone	Cat. No.	Fluorochrome
IgG1, Kappa (isotype control)	BD	MOPC-21	557783	Alexa Fluor 647
AKT (pS473)	CST	D9E	4075	Alexa Fluor 647
BLNK (pY84)	BD	J117-1278	558443	Alexa Fluor 647
Btk (pY551)/Itk (pY511)	BD	24a/BTK (Y551)	558134	Alexa Fluor 647
CD19	TFS	HIB19	45-0199-42	PerCP-Cyanine5.5
Histone H3 (pS10)	CST	D2C8	9716	Alexa Fluor 647
Lck (pY505)	BD	4/LCK-Y505	558577	Alexa Fluor 647
MAPKAPK-2 (pT334)	CST	27B7	4320S	Alexa Fluor 647
MEK1 (pS298)	BD	J114-64	560043	Alexa Fluor 647
NF-κB p65 (pS529)	BD	K10-895.12.50	558422	Alexa Fluor 647
NF-κB p65 (pS536)	CST	93H1	4887	Alexa Fluor 647
p38 MAPK (pT180/Y182)	CST	28B10	4552	Alexa Fluor 647
p44/42 MAPK (pT202/Y204)	CST	E10	4375	Alexa Fluor 647
PLCγ2 (pY759)	BD	K86-689.37	558498	Alexa Fluor 647
Rb (pS807/pS811)	BD	J112-906	558590	Alexa Fluor 647
S6-Ribos. Prot. (pS235/S236)	CST	D57.2.2E	4851	Alexa Fluor 647
SAPK/JNK (pT183/Y185)	CST	G9	9257	Alexa Fluor 647
STAT1 (pY701)	BD	4a	612597	Alexa Fluor 647
STAT3 (pY705)	BD	4/P-STAT3	557815	Alexa Fluor 647
STAT5 (pY694)	BD	47/Stat5(pY694)	612599	Alexa Fluor 647
STAT6 (pY641)	BD	18/P-Stat6	612601	Alexa Fluor 647
SYK (pY525/Y526)	CST	C87C1	12081	Alexa Fluor 647

*BD, Beckton Dickinson Pharmingen; CST, Cell Signaling Technologies; TFS, ThermoFisher Scientific
